# Screen time and obesity prevalence in adolescents: an isotemporal substitution analysis

**DOI:** 10.1186/s12889-024-20639-x

**Published:** 2024-11-12

**Authors:** Dohyun Byun, Yujin Kim, Hajin Jang, Hannah Oh

**Affiliations:** 1 Interdisciplinary Program in Precision Public Health, Department of Public Health Sciences, Graduate School of Korea University, Seoul, Republic of Korea; 2 School of Public Health, University of Pittsburgh, 130 De Soto St, Pittsburgh, PA USA; 3 Division of Health Policy and Management, College of Health Sciences, Korea University, 145 Anam-ro, Seongbuk-gu, Hana Science Building B. Room 358, Seoul, Republic of Korea

**Keywords:** Smartphone, Computer, Television, Internet, Media, Overweight, Children, Adolescent, Teenager, Body mass index

## Abstract

**Background:**

We examined the associations between screen time and adolescent obesity and the associations of reallocating screen time to other activities using isotemporal substitution models. Understanding the association between screen time and obesity may provide additional insights into effective obesity prevention strategies in adolescents.

**Methods:**

We conducted a cross-sectional analysis of 5,180 adolescents (4th and 7th grade students) from the Korean Children and Youth Panel Survey 2018. Participants reported their height, weight, and average time spent watching television, using computer, using smartphone, and other after-school activities. Obesity was defined as BMI-for-age and -sex ≥ 95th percentile based on the 2017 Korean National Growth Charts. We performed multivariable logistic regression to estimate odds ratios (ORs) and 95% confidence intervals (CIs) for the associations between screen time and obesity prevalence, adjusting for potential confounders.

**Results:**

Prolonged smartphone use (≥ 180 vs. <60 m/d: OR [95% CI] = 2.75 [2.06, 3.68]) was associated with a higher obesity prevalence. Television watching (≥ 120 vs. <60 min/d) was positively associated with obesity prevalence among 4th grade students (2.09 [1.51, 2.89]) but the association was not observed among 7th grade students (0.97 [0.63, 1.49]). One-hour increments of any non-screen time activities, with a simultaneous 1-hour decrease in combined screen time, were associated with a lower obesity prevalence (physical activity: 0.75 [0.65, 0.85]; sleeping: 0.69 [0.62, 0.78]; hanging out with friends: 0.80 [0.71, 0.89]; reading: 0.82 [0.69, 0.97]; studying: 0.84 [0.78, 0.90]; chatting with parents: 0.89 [0.88, 0.98]).

**Conclusions:**

Our data suggest that public health strategies that reduce screen time and increase time for non-screen time activities, such as physical activity, may be effective in lowering adolescent obesity prevalence.

**Supplementary Information:**

The online version contains supplementary material available at 10.1186/s12889-024-20639-x.

## Introduction

Adolescents spend a large proportion of their leisure time in front of screens such as televisions, computers, and smartphones [1, 2]. Many studies have shown that prolonged television watching increases the risk of obesity among adolescents [3, 4]. Recent studies also suggested that combined screen time, including time spent using computers and smartphones, is positively associated with obesity prevalence among teenagers [5, 6]. However, most studies did not differentiate the associations by specific types of screen device use [7, 8] and thus it is unclear whether the association varies by the type of screen device use (e.g., smartphone use vs. television watching) among adolescents. While different types of screen devices share similar characteristics such as increasing sedentary time, each type has distinct characteristics that may potentially yield differential effects on adolescent health and behaviors. For example, while televisions provide a limited selection of programs and contents, computers and smartphones offer flexible access to a wider range of contents (e.g., videos, games, webtoons, and social media) that may influence adolescents’ perception on health and nutrition [9–11]. Compared with televisions and computers, smartphones can also be more easily carried around and used without restriction on time and location. For this reason, smartphones are more likely to be used before bedtime [12] and during mealtime [13] and thereby may have a strong influence on sleeping [12, 14] and eating behaviors [10, 15].

Further, while the current guideline recommends less than 2 h/d of screen time for adolescents [16], the guideline does not provide clear recommendations on specific activities that may replace screen time for better health. Given the finite time of 24-hour a day, a decrease in screen time indicates an increase in the time spent on other activities (i.e., substitution effect). The activity choice is likely to differentially influence body weight. The isotemporal substitution models allow addressing these substitutions. Previous findings from isotemporal substitution models suggested the benefit of reallocating sedentary time to physically active time for obesity prevention [17, 18]. However, little is known about the association of reallocating screen time to other daily activities (e.g., physical activity or sleeping) among adolescents.

In this study, we examined the association of combined screen time, as well as specific types of screen device use (television, computer, and smartphone), with obesity prevalence among adolescents, using nationally representative data from South Korea. South Korea is one of the countries with the highest rate of adolescent smartphone use [19]. We also used isotemporal substitution models [20, 21] to examine the associations of reallocating time spent using screen devices to other activities (physical activity, sleeping, hanging out with friends, reading, studying, and chatting with parents). We hypothesized that more modern screen device use, particularly smartphone use, may be associated with higher obesity prevalence and that reallocating screen time for more active behaviors (e.g., physical activity) may lower the obesity prevalence in adolescents.

## Methods

### Study population

We conducted a cross-sectional analysis using data from the first wave of the Korean Children and Youth Panel Survey 2018 (KCYPS 2018). The KCYPS 2018, conducted by the National Youth Policy Institute (NYPI), has two on-going cohorts that began in 2018 among nationally representative samples of 4th grade (elementary school; *n* = 2,607; aged 9–11 years) and 7th grade (1st year in middle school; *n* = 2,590; aged 12–14 years) South Korean students. The participants were selected using a multi-stage stratified cluster sampling based on geographic area. Briefly, 171 elementary schools and 162 middle schools were selected based on the probability proportional to size (PPS) sampling from 17 cities and provinces of Korea. Within each school, a classroom was selected at random. All students in the selected classroom were asked for assent to participate in the study. Among students who agreed to participate, their households (parents or guardians) were contacted to collect consent to participate before the home visit survey. During home visit, the information on behavioral and environmental factors was collected via tablet-assisted in-person interview conducted by trained study staff, following standardized protocols. Parental and household information was collected via separate interview of participant’s parent or guardian. The survey was designed and developed through focus group and pilot studies that assessed the quality and reliability of responses from the target population. The same survey methods and procedures were used in both cohorts (4th and 7th grade). All participants and their parents (or guardians) provided informed consent and the study was approved by the Ethics Committee of the National Youth Policy Institute and Korea University (IRB-2022-0037).

Among 5,197 KCYPS 2018 participants (2,607 4th grade and 2,590 7th grade students), we excluded participants with missing information on weight or height (*n* = 17). In total, 5,180 participants (2,594 4th grade and 2,586 7th grade students) were included in the current analysis (participant flowchart in Supplementary Fig. 1).

### Screen time and other after-school activities

Participants were asked to report the average time they spent on each of the following activities on usual weekdays and weekends during the past academic semester: using smartphones for leisure, using computers for leisure, watching television for leisure, chatting with parents or caregivers, studying at private academies or tutoring, watching lectures online or on television, attending after-school classes, doing homework or studying alone, reading for leisure (excluding reading textbooks), exercising or performing physical activity after school, and hanging out with friends. The responses were reported in categories: never, < 30 min, 30 min to < 1 h, 1 h to < 2 h, 2 h to < 3 h, 3 h to < 4 h, and ≥ 4 h. For each activity, we assigned the midpoint value to each category (the width of open-ended extreme category was assumed to be the same as that of adjacent category) and estimated average daily duration using the following formula: (weekday duration × 5 + weekend duration × 2)/7. For each participant, combined screen time was estimated as the sum of time spent watching television, using computers, and using smartphones during leisure time. For easier interpretation, each screen time variable was categorized into the 60-minute intervals. After collapsing extreme intervals with scarce data, the following categories were used in the analyses: combined screen time (< 120, 120 to < 180, 180 to < 240, and ≥ 240 m/d); smartphone use (< 60, 60 to < 120, 120 to < 180, ≥180 min/d); computer use (< 60, 60 to < 120, ≥120 min/d); and television watching (< 60, 60 to < 120, ≥120 min/d). Duration of studying at private academies or tutoring, watching lectures online or on television, attending after-school classes, and doing homework or studying were summed to represent combined study time. Participants were also asked to report the usual time going to bed at night and getting up in the morning, separately for weekdays and weekends. Based on the response, we estimated average daily sleep duration. For an isotemporal substitution analysis, we estimated the average hour spent on each activity during the 24-hour day by assuming that the activities assessed in the study is an exhaustive list that participants performed during an average day. We also assumed that the contributions of other daily activities that were not assessed in the study would be minimal. Because the duration of activities did not exactly add up to 24 h in some participants, we first calculated the proportion that each activity (using smartphones, using computers, watching television, performing physical activity, hanging out with friends, chatting with parents, studying, reading) contributed to participant’s summed duration of activities and then multiplied the proportion by participant’s waking hours (24 h minus sleeping and school hours). In the end, the activity times added up to 24 h within each participant, as indicated by the following Eq. (24 h = time using smartphones + time using computers + time watching televisions + time performing physical activity + sleeping time + time hanging out with friends + reading time + studying time + time chatting with parents + time at school).

### Obesity

Participants were asked to report their current weight and height. Body mass index (BMI) was calculated as weight (kg) divided by height squared (m^2^). Obesity was defined as those with BMI-for-age and -sex ≥ 95th percentile based on the 2017 Korean National Growth Charts for children and adolescents [22].

### Covariates

During interview, participants were asked to report their sex (boy or girl), date of birth, and academic performance (5 Likert scale: very poor, poor, fair, good, very good). Perceived parental control was assessed using the Korean Version of Parents as Social Context Questionnaire for Adolescents (PSCQ_KA), which has previously been validated [23]. Perceived parental psychological control score (range 4–16) was calculated by summing the scores from the following 4 questions related to parental psychological control (“My parents always push me to do something.”; “My parents tell me what I do.”; “My parents think that when they do something, their way is the only way to do it.”; “My parents say “no” to everything.”). Each question was responded in the 4 Likert scale (score 1–4). Higher scores indicate stronger perceived parental psychological control. For each participant, a parent (or a guardian) was separately interviewed to collect parental and household information, including a list of household members, mother’s and father’s highest education, and average monthly household income.

### Statistical analysis

We performed multivariable logistic regression to estimate odds ratios (ORs) and 95% confidence intervals (CIs) for the association between screen time and obesity prevalence in each cohort (4th and 7th grade students), as well as in the pooled population. Multivariable models included potential confounders: grade, sex, parental co-residence, parents’ highest education level, monthly household income, academic performance, and perceived parental control. Additional adjustment for parents’ occupation and participant’s depressive symptom score did not change the OR estimates and thus these variables were not included in the final models. To account for clustering effect of students, we additionally adjusted for school in sensitivity analyses. Results did not change and thus school was not included in the final models (Supplementary Table 1). We tested for linear trends using the Wald test for continuous exposure variables (h/d). We also examined effect modification by school grade (4th grade vs. 7th grade) and sex (boy vs. girl) because susceptibility and risk factors for obesity may vary by these variables. We tested for interaction using the Wald test for product terms. Next, we performed isotemporal substitution analyses. The isotemporal substitution models, by definition, estimate the association of reallocating one activity type to another activity type for the same amount of time (e.g., reallocating time spent on television watching to physical activity) [20, 21]. In isotemporal substitution models, we included all activity time variables that contributed to participant’s 24-hour time composition, except for screen time variables (the activity being displaced), as follows: Logit (obesity) = β_0_ + (β_1_) time performing physical activity + (β_2_) sleeping time + (β_3_) time hanging out with friends + (β_4_) reading time + (β_5_) studying time + (β_6_) time chatting with parents + covariates. Time at school was not included in the model because it was the same for all participants in the same grade. In these models, the OR indicates the association of 1-hour increment in the corresponding activity time (with simultaneous 1-hour decrement in screen time), while holding the other activity times constant.

All statistical tests were 2-sided with a 5% type I error rate. Bonferroni correction was performed to adjust for multiple testing. All analyses were conducted using SAS version 9.4 (SAS Institute).

## Results

### Study population

The obesity prevalence was 9.6% (10.4% 4th grade; 8.9% 7th grade) and mean combined screen time was 3.0 h/d (2.6 h/d 4th grade; 3.4 h/d 7th grade) in the pooled population (Table 1). Compared with 7th grade students, the 4th grade students were more likely to have a longer duration of television watching (1.1 vs. 0.8 h/d) and shorter durations of smartphone use (1.3 vs. 1.9 h/d) and computer use (0.3 vs. 0.7 h/d). Students with prolonged combined screen time (≥ 240 vs. <120 min/d) were more likely to be boys and have lower household income, poor academic performance, and lower parental education, and were less likely to live with both parents (Supplementary Table 2). They also had longer time hanging out with friends and shorter time sleeping, performing physical activity, chatting with parents, studying, and reading.

**Table 1:** Characteristics of study participants in the KCYPS 2018

Characteristics	Pooled population (*n* = 5180)	4th grade students (*n* = 2594)	7th grade students (*n* = 2586)
	*N* (%) or mean (SD)		
**Sex**			
Boys	2710 (52.3)	1306 (50.3)	1404 (54.3)
Girls	2470 (47.7)	1288 (49.7)	1182 (45.7)
**Obesity prevalence** ^**a**^	498 (9.6)	269 (10.4)	229 (8.9)
**Parental co-residence**			
None or one parent	452 (8.7)	208 (8.0)	244 (9.4)
Both parents	4728 (91.3)	2386 (92.0)	2342 (90.6)
**Parents’ highest education**			
High school or lower	1185 (22.9)	507 (19.6)	678 (26.2)
College or higher	3995 (77.1)	2087 (80.4)	1908 (73.8)
**Monthly household income, KRW**			
< 2,000,000	309 (6.0)	149 (5.7)	160 (6.2)
2,000,000 to < 4,000,000	1352 (26.1)	704 (27.1)	648 (25.1)
4,000,000 to < 6,000,000	2044 (39.4)	1027 (39.6)	1017 (39.3)
6,000,000 to < 8,000,000	869 (16.8)	421 (16.2)	448 (17.3)
≥ 8,000,000	606 (11.7)	293 (11.4)	313 (12.1)
**Academic performance**			
Poor or fair	2090 (40.3)	779 (30.0)	1311 (50.7)
Good	2841 (54.9)	1685 (65.0)	1156 (44.7)
Missing	249 (4.8)	130 (5.0)	119 (4.6)
**Perceived parental control score** ^**b**^	8.6 (2.6)	8.4 (2.6)	8.7 (2.6)
**Average duration of daily activities, h/d**			
Combined screen time	3.0 (1.6)	2.6 (1.6)	3.4 (1.6)
Using smartphone	1.6 (1.2)	1.3 (1.1)	1.9 (1.2)
Using computer	0.5 (0.8)	0.3 (0.5)	0.7 (0.9)
Watching television	0.9 (0.8)	1.1 (0.9)	0.8 (0.7)
Performing physical activity	1.0 (0.8)	1.1 (0.8)	0.8 (0.7)
Sleeping	8.8 (1.0)	9.3 (0.8)	8.4 (0.9)
Hanging out with friends	1.3 (0.9)	1.3 (0.9)	1.3 (1.0)
Reading	0.6 (0.6)	0.7 (0.7)	0.5 (0.5)
Studying	3.5 (1.6)	3.7 (1.5)	3.3 (1.6)
Chatting with parents	1.6 (1.0)	1.7 (1.1)	1.4 (0.9)

*Abbreviation* KCYPS, Korean Children and Youth Panel Survey; KRW: South Korean Won; SD, standard deviation

^a^ Obesity was defined as age- and sex-specific body mass index ≥ 95th percentile based on the 2017 Korean National Growth Charts for children and adolescents

^b^ Perceived parental psychological control score (range 4–16) was calculated by summing the scores from the following 4 questions related to parental psychological control (My parents always push me to do something; My parents tell me what I do; My parents think that when they do something, their way is the only way to do it; My parents say “no” to everything). Each question had a score that ranged 1–4

### Association between screen time and obesity

Table 2 presents the association between screen time and obesity prevalence. Prolonged combined screen time (≥ 240 vs. <120 m/d) was statistically significantly associated with a higher obesity prevalence in all groups (OR [95% CI] = 2.42 [1.83, 3.20] pooled population; 2.76 [1.95, 3.92] 4th grade; 1.79 [1.11, 2.89] 7th grade). When we examined the associations with specific types of screen device use, prolonged smartphone use (≥ 180 vs. <60 m/d) was positively associated with obesity prevalence in all groups (OR [95% CI] = 2.75 [2.06, 3.68] pooled; 3.59 [2.42, 5.32] 4th grade; 2.00 [1.28, 3.12] 7th grade). Prolonged television watching (≥ 120 vs. <60 min/d) was positively associated with obesity prevalence among the 4th grade students (OR [95% CI] = 2.09 [1.51, 2.89]) but the positive association was not observed among the 7th grade students (0.97 [0.63, 1.49]; p-interaction = 0.02). Among the 4th grade students, computer use was only marginally significantly associated with a higher obesity prevalence (OR [95% CI] = 1.73 [0.93, 3.21]; p-trend = 0.05). Most associations remained statistically significant after Bonferroni correction for multiple testing.

**Table 2:** Odds ratios (ORs) and 95% confidence intervals (CIs) for the associations between screen time and obesity prevalence

Screen time	Pooled population		Stratified by grade in school				
			4th grade students		7th grade students		
	Cases / *N*	OR (95% CI) ^a^	Cases / *N*	OR (95% CI) ^b^	Cases / *N*	OR (95% CI) ^b^	*p*-int ^c^
**Combined screen time, m/d**							
< 120	85 / 1464	1.00 (ref)	63 / 1062	1.00 (ref)	22 /402	1.00 (ref)	
120 to < 180	87 / 1028	1.54 (1.13, 2.11) ^f^	51 / 556	1.49 (1.01, 2.20)	36 / 472	1.39 (0.80, 2.41)	
180 to < 240	106 / 970	2.05 (1.51, 2.78) ^f^	64 / 441	2.42 (1.67, 3.52) ^f^	42 / 529	1.40 (0.82, 2.39)	
≥ 240	220 / 1718	2.42 (1.83, 3.20) ^f^	91 / 535	2.76 (1.95, 3.92) ^f^	129 / 1183	1.79 (1.11, 2.89)	
p-trend ^d^		< 0.0001 ^f^		< 0.0001 ^f^		0.01	0.21
**Smartphone use, m/d**							
< 60	122 / 1925	1.00 (ref)	90 / 1382	1.00 (ref)	32 / 543	1.00 (ref)	
60 to < 120	138 / 1417	1.69 (1.30, 2.19) ^f^	71 / 662	1.62 (1.17, 2.26) ^f^	67 / 755	1.53 (0.99, 2.39)	
120 to < 180	115 / 968	2.24 (1.69, 2.97) ^f^	57 / 323	2.84 (1.97, 4.08) ^f^	58 / 645	1.57 (1.00, 2.47)	
≥ 180	123 / 870	2.75 (2.06, 3.68) ^f^	51 / 227	3.59 (2.42, 5.32) ^f^	72 / 643	2.00 (1.28, 3.12) ^f^	
p-trend ^d^		< 0.0001 ^f^		< 0.0001 ^f^		0.004 ^f^	0.05
**Computer use, m/d**							
< 60	381 / 4140	1.00 (ref)	235 / 2374	1.00 (ref)	146 / 1766	1.00 (ref)	
60 to < 120	59 / 607	0.95 (0.70, 1.29)	20 / 146	1.30 (0.79, 2.14)	39 / 461	0.76 (0.52, 1.12)	
≥ 120	58 / 433	1.26 (0.92, 1.74)	14 / 74	1.73 (0.93, 3.21)	44 / 359	1.08 (0.73, 1.57)	
p-trend ^d^		0.26		0.05		0.99	0.18
**Television watching, m/d**							
< 60	278 / 3217	1.00 (ref)	130 / 1531	1.00 (ref)	148 / 1686	1.00 (ref)	
60 to < 120	125 / 1273	1.16 (0.93, 1.45)	72 / 689	1.25 (0.92, 1.70)	53 / 584	1.07 (0.77, 1.50)	
≥ 120	95 / 690	1.53 (1.19, 1.98) ^f^	67 / 374	2.09 (1.51, 2.89) ^f^	28 / 316	0.97 (0.63, 1.49)	
p-trend ^d^		0.001 ^f^		< 0.0001 ^f^		0.96	0.02

*Abbreviation* OR, Odds Ratios; CI, Confidence intervals

^a^ Adjusted for grade (4th; 7th grade), sex (boy; girl), parental co-residence (none or one parent; both parents), parents’ highest education (high school or lower; college or higher), monthly household income (< 2,000,000; 2,000,000 to < 4,000,000; 4,000,000 to < 6,000,000; 6,000,000 to < 8,000,000; ≥8,000,000 KRW), academic performance (poor or fair; good; missing), and perceived parental control score (continuous)

^b^ Adjusted for sex (boy; girl), parental co-residence (none or one parent; both parents), parents’ highest education (high school or lower; college or higher), monthly household income (< 2,000,000; 2,000,000 to < 4,000,000; 4,000,000 to < 6,000,000; 6,000,000 to < 8,000,000; ≥8,000,000 KRW), academic performance (poor or fair; good; missing), and perceived parental control score (continuous)

^c^ P-interaction was estimated using the Wald test for the production term between screen time and grade

^d^ P-trend was estimated using the Wald test for continuous screen time variables

^f^ Statistical significance at Bonferroni-corrected α level of 0.0125 (4 exposures)

### Isotemporal substitution analysis

**Fig. 1:**
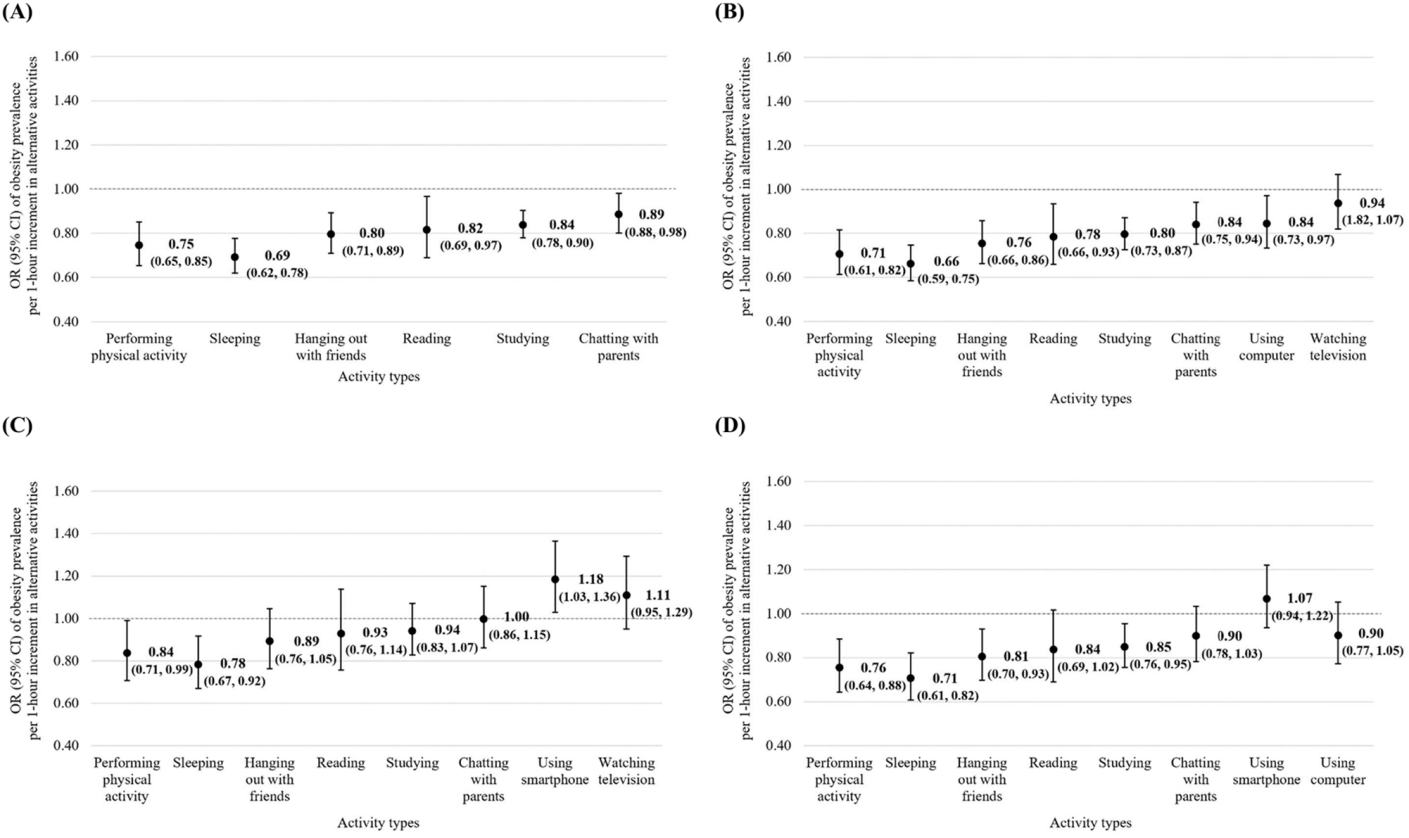
Odds ratios (ORs) and 95% confidence intervals (CIs) of obesity prevalence associated with substituting 1-hour of screen time for 1-hour of non-screen time activities in the pooled population. The graphs show the ORs and 95% CIs of obesity prevalence associated with increasing 1-hour of each non-screen time activity while decreasing 1-hour of (**A**) combined screen time, (**B**) smartphone use, (**C**) computer use, and (**D**) television watching in the pooled population. Models adjusted for school grade (4th, 7th grade), sex (boy, girl), parental co-residence (none or one parent, both parents), parents’ highest education (high school or lower, college or higher), monthly household income (< 2,000,000; 2000,000 to < 4,000,000; 4,000,000 to < 6,000,000; 6,000,000 to < 8,000,000; ≥8,000,000 KRW), academic performance (poor or fair, good, missing), perceived parental control (score 4–16, continuous), and all non-screen time activities (performing physical activity, sleeping, hanging out with friends, reading, studying, chatting with parent)

Figure 1 shows the results from isotemporal substitution analysis in the pooled population. One-hour increments of any non-screen time activities, with a simultaneous 1-hour decrease in combined screen time, were associated with a lower obesity prevalence (physical activity: 0.75 [0.65, 0.85]; sleeping: 0.69 [0.62, 0.78]; hanging out with friends: 0.80 [0.71, 0.89]; reading: 0.82 [0.69, 0.97]; studying: 0.84 [0.78, 0.90]; chatting with parents: 0.89 [0.88, 0.98]; Fig. 1A). Similarly, substituting 1-hour of smartphone use for non-screen time activities (physical activity: 0.71 [0.61, 0.82]; sleeping: 0.66 [0.59, 0.75]; hanging out with friends: 0.76 [0.66, 0.86]; reading: 0.78 [0.66, 0.93]; studying: 0.80 [0.73, 0.87]; chatting with parents: 0.84 [0.75, 0.94]) or computer use (0.84 [0.73, 0.97]) were inversely associated with obesity prevalence (Fig. 1B). Substituting 1-hour of computer use for physical activity (0.84 [0.71, 0.99]) or sleeping (0.78 [0.67, 0.92]) were associated with lower obesity prevalence, whereas substituting for smartphone use was associated with a higher obesity prevalence (1.18 [1.03, 1.36]; Fig. 1C). For television watching, physical activity (0.76 [0.64, 0.88]), sleeping (0.71 [0.61, 0.82]), hanging out with friends (0.81 [0.70, 0.93]), and studying (0.85 [0.76, 0.95]) were inversely associated with obesity prevalence (Fig. 1D). Similar results were observed when we separately examined the associations among the 4th (Supplementary Fig. 2) and 7th grade students (Supplementary Fig. 3).

In the stratified analysis by sex, similar patterns of associations were observed in both boys and girls (Supplementary Table 3; Supplementary Figs. 4–5).

## Discussion

In this cross-sectional analysis of nationally representative samples of Korean adolescents, we observed that prolonged screen time, particularly smartphone use, was associated with a higher prevalence of obesity. In isotemporal substitution analyses, reallocating screen time to any non-screen time activities, particularly performing physical activity and sleeping, were associated with a lower obesity prevalence in Korean adolescents. Our data suggest potential benefit of substituting screen time for non-screen time activities among Korean adolescents.

Our findings of the positive association between screen time and obesity are consistent with those from previous studies [5–7]. In our analysis, we further demonstrated that the positive association persisted after adjustment for potential confounders, including parental and socioeconomic factors, supporting the robustness of our results. During screen time, adolescents are likely to be exposed to advertisements of energy-dense foods [24, 25] and various contents that deliver misleading messages on food and nutrition [26–29]. Unhealthy food perception may lead to unhealthy food choices and eating behaviors [30]. Eating during screen time can also promote snacking, particularly the consumption of obesogenic, ultra-processed foods (e.g., ready-meals, instant foods) [10, 31], and “mindless eating” (eating without acknowledgement of the quantity and quality of foods) [32], leading to overeating and extra calorie consumption [33]. In addition, because screen devices are often used in lying or sitting positions, screen time may increase sedentary time, resulting in lower daily calorie expenditure and dysregulated metabolism [6, 34]. Prolonged screen time may also disrupt sleep duration and quality [6, 35–38], leading to altered appetite and metabolism [39]. Participants with prolonged screen time are also likely to have shorter time left for other beneficial activities such as physical activity.

In the present study, we further investigated the associations with three different types of screen device use (television, computer, and smartphone). We observed that the positive association with obesity was most pronounced for smartphone use, which also accounted for the largest proportion of combined screen time among adolescents. In addition, our isotemporal substitution analysis showed that substituting the time spent using smartphones for using computers was also inversely associated with obesity prevalence, suggesting a possible variation in associations among different screen device use. The variation may be due to the differences in content type (e.g., games, video, social media) and environmental setting (e.g., day vs. night, bedroom vs. outdoor, alone vs. with friends) that adolescents are commonly exposed to while using specific screen devices. For example, computers may be more likely to be used for information search and retrieval, while smartphones may be more likely to be used for accessing entertainment contents (e.g., games, video). A previous study has shown that using screen devices mainly for entertainment contents is associated with more frequent intake of obesogenic foods, such as sugar-sweetened beverages, compared with using mainly for education and information search [10]. In another study, prolonged leisure-time internet use but not study-time internet use was associated with more frequent intakes of fast foods and sugar-sweetened beverages [11], suggesting that the associations with dietary behaviors may vary by the content type. Further, with widespread algorithm-based advertising in digital platforms, the types of advertisement being exposed and the accuracy of information obtained may also vary depending on the contents commonly accessed while using screen devices. The associations may also vary depending on the time of the day and the place being used. Screen use during mealtime and before bedtime are more likely to influence eating and sleeping behaviors than screen use outside of meal- or bedtime [15, 37, 40]. The time of the day is also likely to influence appetite and cravings. Compared with other screen devices, smartphone is the most portable device and thus is more likely to be used during mealtime and before bedtime [41]. Smartphone use during mealtime can promote snacking and mindless eating, leading to consumption of extra calories [32, 33]. Smartphone use before bedtime can disrupt sleep patterns [37], leading to hormone imbalance and appetite dysregulation [39]. Dietary environment and food options during screen time are also likely to differ when using screen devices at home vs. during transportation. However, our data lack information on the time, content, and place of screen device use and thus we were not able to examine the associations after accounting for these factors. Further studies are needed to clarify the variation in contents and environmental settings being exposed while using different screen devices.

In our study, we also observed that reallocating time spent using screen devices to performing physical activity and sleeping were associated with a lower obesity prevalence among both 4th and 7th grade students. Spending more time on non-sedentary activities (e.g., playing sports) may increase energy expenditure and lower obesity prevalence. Although sleeping is a sedentary behavior, sufficient sleep helps regulate metabolism and appetite [42, 43]. Compared with screen time, sleeping is also likely to contribute to lower energy consumption because there is no extra calorie consumption while sleeping. We also observed that substituting combined screen time for other non-screen time activities, such as reading, studying, and chatting with parents, were inversely associated with obesity prevalence. Consistent with our findings, a previous study reported that substituting combined screen time for other sedentary activities, such as reading, talking, and listening to music, was associated with lower BMI among Chinese 1st to 3rd grade students [44]. Regardless of the type of activities replacing screen time, reducing screen time itself is likely to reduce obesity prevalence by decreasing indiscriminate exposure to unhealthy, obesogenic contents (e.g., advertisements of unhealthy foods). Adolescents are also likely to consume less energy from drinks and solid foods while reading and talking, compared with while watching screens [45, 46]. Compared with screen time activities, reading and talking are also likely to involve more active brain engagement [47]. Further, positive relationship with parents via frequent, intimate communications has shown to be associated with a lower obesity risk [48]. Using isotemporal substitution models, our findings provide additional insights for future public health recommendations.

We acknowledge that this study has several limitations. First, because of the cross-sectional design of our study, we cannot confirm the temporal relationship between screen time and obesity. The relationship between screen time and obesity may be bi-directional [3] and thus longitudinal studies are needed to confirm our results. Second, given the self-reported data, our analysis is also subject to measurement errors. For screen time and other activity variables, participants were asked to report the average time spent on each activity during the past semester and thus the accurate recall required good memory. Additionally, because the survey was conducted via face-to-face interview, the data are also subject to social desirability bias that may lead to under-reporting of screen time and obesity. Further studies may be needed to investigate the associations using more objective measurements (e.g., actigraph). Lastly, in the isotemporal substitution analysis, we assumed that the contributions of other daily activities that were not assessed in the study would be minimal. Our results may include measurement errors if our assumption is not valid. Further studies are needed to confirm our results using more detailed data on daily activities.

Despite the limitations, this study has important strengths. We increased the generalizability of our study findings by using nationally representative samples of Korean adolescents. However, our results may not be generalizable to other populations of different ages and ethnicity if the contents and purpose of screen device use are different from those of our study population. Korea has one of the highest adolescent smartphone usage rate, with widespread use of online media platforms, such as YouTube and Instagram [19]. Adolescents’ screen media literacy may also influence their susceptibility to negative health effects of media contents. Further, while most studies evaluated combined screen time [17, 18, 49], our study also separately examined the associations of television watching, computer use, and smartphone use and the associations of substituting screen time for various non-screen time activities.

## Conclusions

In summary, we observed that prolonged screen time, particularly smartphone use, was positively associated with obesity prevalence among Korean adolescents. Reallocating the time spent using screen devices to non-screen time activities, such as performing physical activity and sleeping, was associated with a lower obesity prevalence. As adolescent obesity increases the risks of adult obesity and various chronic diseases, it is important to understand risk factors for obesity in contemporary adolescents. This study provides important insights into the negative health consequences of prolonged screen time and informs public health strategies to reduce obesity prevalence in adolescents. Given the worldwide increase in screen time and obesity prevalence among adolescents, public health strategies should focus on encouraging adolescents to engage in physical activity while decreasing screen device use. More efforts should also focus on providing appropriate guidance for developing healthy screen use behavior in adolescents and limiting access to unhealthy food marketing and inaccurate health information in screen media.

## Electronic supplementary material

Below is the link to the electronic supplementary material.

Supplementary Material 1

## Data Availability

Data described in the manuscript is made publicly and freely available without restriction at National Youth Policy Institute (NYPI) Youth and Children Data Archive (https://www.nypi.re.kr/archive/mps).
