# Supplementary material for: Screen time and obesity prevalence in adolescents: an isotemporal substitution analysis
Source: BMC Public Health. 2024 Nov 12;24:3130. doi: 10.1186/s12889-024-20639-x (PMC11555804; doi:10.1186/s12889-024-20639-x)
Supplement: Supplementary file 1 — Supplementary Material 1 [file 12889_2024_20639_MOESM1_ESM.docx]

**Supplementary Table 1. Odds ratios (ORs) and 95% confidence intervals (CIs) for the associations between screen time and obesity prevalence, after additionally adjustment for school**

| **Screen time** | **Pooled population** | | **Stratified by grade in school** | | | | |
| --- | --- | --- | --- | --- | --- | --- | --- |
|  |  |  | **4^th^ grade students** | | **7^th^ grade students** | |  |
|  | **Cases / N** | **OR (95% CI) ^a^** | **Cases / N** | **OR (95% CI) ^b^** | **Cases / N** | **OR (95% CI) ^b^** | **p-int ^c^** |
| **Combined screen time, m/d** |  |  |  |  |  |  |  |
| <120 | 85 / 1464 | 1.00 (ref) | 63 / 1062 | 1.00 (ref) | 22 /402 | 1.00 (ref) |  |
| 120 to <180 | 87 / 1028 | 1.54 (1.12, 2.11) ^e^ | 51 / 556 | 1.49 (1.01, 2.20) | 36 / 472 | 1.39 (0.80, 2.42) |  |
| 180 to <240 | 106 / 970 | 2.05 (1.51, 2.78) ^e^ | 64 / 441 | 2.42 (1.67, 3.52) ^e^ | 42 / 529 | 1.41 (0.82, 2.41) |  |
| **≥**240 | 220 / 1718 | 2.41 (1.82, 3.18) ^e^ | 91 / 535 | 2.76 (1.95, 3.92) ^e^ | 129 / 1183 | 1.80 (1.12, 2.89) |  |
| p-trend ^d^ |  | <0.0001 ^e^ |  | <0.0001 ^f^ |  | 0.01 | 0.22 |
| **Smartphone use, m/d** |  |  |  |  |  |  |  |
| **<**60 | 122 / 1925 | 1.00 (ref) | 90 / 1382 | 1.00 (ref) | 32 / 543 | 1.00 (ref) |  |
| 60 to <120 | 138 / 1417 | 1.69 (1.30, 2.19) ^e^ | 71 / 662 | 1.62 (1.17, 2.26) ^e^ | 67 / 755 | 1.54 (0.99, 2.40) |  |
| 120 to <180 | 115 / 968 | 2.24 (1.69, 2.96) ^e^ | 57 / 323 | 2.83 (1.97, 4.08) ^e^ | 58 / 645 | 1.57 (1.00, 2.47) |  |
| **≥**180 | 123 / 870 | 2.74 (2.05, 3.67) ^e^ | 51 / 227 | 3.58 (2.41, 5.32) ^e^ | 72 / 643 | 2.00 (1.28, 3.13) ^e^ |  |
| p-trend ^d^ |  | <0.0001 ^e^ |  | <0.0001 ^f^ |  | 0.004 ^e^ | 0.05 |
| **Computer use, m/d** |  |  |  |  |  |  |  |
| <60 | 381 / 4140 | 1.00 (ref) | 235 / 2374 | 1.00 (ref) | 146 / 1766 | 1.00 (ref) |  |
| 60 to <120 | 59 / 607 | 0.95 (0.70, 1.29) | 20 / 146 | 1.30 (0.79, 2.14) | 39 / 461 | 0.76 (0.52, 1.12) |  |
| **≥**120 | 58 / 433 | 1.26 (0.91, 1.74) | 14 / 74 | 1.72 (0.93, 3.19) | 44 / 359 | 1.07 (0.73, 1.57) |  |
| p-trend ^d^ |  | 0.27 |  | 0.05 |  | 0.97 | 0.18 |
| **Television watching, m/d** |  |  |  |  |  |  |  |
| <60 | 278 / 3217 | 1.00 (ref) | 130 / 1531 | 1.00 (ref) | 148 / 1686 | 1.00 (ref) |  |
| 60 to <120 | 125 / 1273 | 1.16 (0.92, 1.45) | 72 / 689 | 1.25 (0.92, 1.69) | 53 / 584 | 1.08 (0.77, 1.50) |  |
| **≥**120 | 95 / 690 | 1.53 (1.19, 1.97) ^e^ | 67 / 374 | 2.08 (1.50, 2.88) ^e^ | 28 / 316 | 0.97 (0.63, 1.49) |  |
| p-trend ^d^ |  | 0.001 ^e^ |  | <0.0001 ^e^ |  | 0.96 | 0.02 |

Abbreviation: OR, Odds Ratios; CI, Confidence intervals.

^a^ Adjusted for grade (4^th^; 7^th^ grade), sex (boy; girl), parental co-residence (none or one parent; both parents), parents’ highest education (high school or lower; college or higher), monthly household income (<2,000,000; 2,000,000 to <4,000,000; 4,000,000 to <6,000,000; 6,000,000 to <8,000,000; ≥8,000,000 KRW), academic performance (poor or fair; good; missing), perceived parental control score (continuous), and school ID (continuous).

^b^ Adjusted for sex (boy; girl), parental co-residence (none or one parent; both parents), parents’ highest education (high school or lower; college or higher), monthly household income (<2,000,000; 2,000,000 to <4,000,000; 4,000,000 to <6,000,000; 6,000,000 to <8,000,000; ≥8,000,000 KRW), academic performance (poor or fair; good; missing), perceived parental control score (continuous), and school ID (continuous).

^c^ P-interaction was estimated using the Wald test for the production term between screen time and grade.

^d^ P-trend was estimated using the Wald test for continuous screen time variables.

^e^ Statistical significance at Bonferroni-corrected α level of 0.0125 (4 exposures)

**Supplementary Table 2.** **Characteristics of study participants according to combined screen time categories in the pooled population of the KCYPS 2018**

| **Characteristics** | **Combined screen time (min/d)** | | | | | | | |
| --- | --- | --- | --- | --- | --- | --- | --- | --- |
|  | **<120** | | **120 to <180** | | **180 to <240** | | **≥240** | |
|  | **N (%)** | | | | | | | |
| **Grade** |  | |  | |  | |  | |
| 4^th^ grade elementary students | 1062 (72.5) | | 556 (54.1) | | 441 (45.5) | | 535 (31.1) | |
| 7^th^ grade middle school students | 402 (27.5) | | 472 (45.9) | | 529 (54.5) | | 1183 (68.9) | |
| **Sex** |  | |  | |  | |  | |
| Boys | 702 (48.0) | | 527 (51.3) | | 510 (52.6) | | 971 (56.5) | |
| Girls | 762 (52.1) | | 501 (48.7) | | 460 (47.4) | | 747 (43.5) | |
| **Obesity ^a^** | 85 (5.8) | | 87 (8.5) | | 106 (10.9) | | 220 (12.8) | |
| **Parental co-residence** |  | |  | |  | |  | |
| None or one parent | 88 (6.0) | | 78 (7.6) | | 79 (8.1) | | 207 (12.1) | |
| Both parents | 1376 (94.0) | | 950 (92.4) | | 891 (91.9) | | 1511 (87.9) | |
| **Parents’ highest education** |  | |  | |  | |  | |
| High school or lower | 207 (14.2) | | 201 (19.6) | | 235 (24.2) | | 542 (31.6) | |
| College or higher | 1257 (85.9) | | 827 (80.4) | | 735 (75.8) | | 1176 (68.4) | |
| **Monthly household income (KRW)** |  | |  | |  | |  | |
| <2,000,000 | 62 (4.2) | | 50 (4.9) | | 50 (5.1) | | 147 (8.6) | |
| 2,000,000 to <4,000,000 | 311 (21.2) | | 266 (25.9) | | 254 (26.2) | | 521 (30.3) | |
| 4,000,000 to <6,000,000 | 604 (41.3) | | 397 (38.6) | | 410 (42.3) | | 633 (36.8) | |
| 6,000,000 to <8,000,000 | 275 (18.8) | | 186 (18.1) | | 154 (15.9) | | 254 (14.8) | |
| ≥8,000,000 | 212 (14.5) | | 129 (12.5) | | 102 (10.5) | | 163 (9.5) | |
| **Academic performance** |  | |  | |  | |  | |
| Poor or fair | 430 (29.4) | | 371 (36.1) | | 420 (43.3) | | 869 (50.6) | |
| Good | 964 (65.9) | | 619 (60.2) | | 515 (53.1) | | 743 (43.2) | |
| Missing | 70 (4.8) | | 38 (3.7) | | 35 (3.6) | | 106 (6.2) | |
|  | **Mean** | **SD** | **Mean** | **SD** | **Mean** | **SD** | **Mean** | **SD** |
| **Parental psychological control score ^b^** | 8.5 | 2.6 | 8.5 | 2.5 | 8.7 | 2.6 | 8.7 | 2.6 |
| **Average duration of daily activities (h/d)** |  |  |  |  |  |  |  |  |
| Using smartphone | 0.7 | 0.6 | 1.4 | 0.9 | 1.9 | 1.1 | 2.3 | 1.2 |
| Using computer | 0.1 | 0.3 | 0.3 | 0.5 | 0.5 | 0.7 | 0.9 | 1.0 |
| Watching television | 0.6 | 0.5 | 0.8 | 0.7 | 1.0 | 0.8 | 1.2 | 0.9 |
| Combined screen time | 1.5 | 0.8 | 2.5 | 0.9 | 3.3 | 1.0 | 4.4 | 1.4 |
| Sleeping | 9.1 | 0.9 | 8.9 | 0.9 | 8.8 | 0.9 | 8.5 | 1.0 |
| Performing physical activity | 1.1 | 0.9 | 1.0 | 0.8 | 0.9 | 0.8 | 0.8 | 0.7 |
| Hanging out with friends | 1.2 | 1.1 | 1.3 | 0.9 | 1.3 | 0.9 | 1.4 | 0.9 |
| Chatting with parents | 1.9 | 1.2 | 1.7 | 1.0 | 1.5 | 0.9 | 1.3 | 0.8 |
| Studying | 4.3 | 1.6 | 3.8 | 1.4 | 3.3 | 1.3 | 2.7 | 1.4 |
| Reading | 0.9 | 0.8 | 0.6 | 0.6 | 0.5 | 0.5 | 0.4 | 0.4 |

Abbreviation: KCYPS, Korean Children and Youth Panel Survey; KRW: South Korean Won; SD, standard deviation.

^a^ Obesity was defined as age- and sex-specific body mass index ≥95^th^ percentile based on the 2017 Korean National Growth Charts for children and adolescents

^b^ Perceived parental psychological control score (range 4-16) was calculated by summing the scores from the following 4 questions related to parental psychological control (My parents always push me to do something; My parents tell me what I do; My parents think that when they do something, their way is the only way to do it; My parents say “no” to everything). Each question had a score that ranged 1-4.

**Supplementary Table 3. Odds Ratios (ORs) and 95% confidence intervals (CIs) for the associations between screen time and obesity prevalence, stratified by sex**

| **Screen time** | **Stratified by sex** | | | | |
| --- | --- | --- | --- | --- | --- |
|  | **Male** | | **Female** | |  |
|  | **Cases / N** | **OR (95% CI) ^b^** | **Cases / N** | **OR (95% CI) ^b^** | **p-int ^c^** |
| **Combined screen time, m/d** |  |  |  |  |  |
| <120 | 54 / 702 | 1.00 (ref) | 31 / 762 | 1.00 (ref) |  |
| 120 to <180 | 54 / 527 | 1.35 (0.91, 2.01) | 33 / 501 | 1.56 (0.94, 2.60) |  |
| 180 to <240 | 68 / 510 | 1.81 (1.24, 2.65) ^f^ | 38 / 460 | 1.86 (1.13, 3.06) |  |
| **≥**240 | 149 / 971 | 2.04 (1.45, 2.86) ^f^ | 71 / 747 | 1.96 (1.25, 3.06) ^f^ |  |
| p-trend ^d^ |  | <0.0001 ^f^ |  | 0.003 | 0.93 |
| **Smartphone use, m/d** |  |  |  |  |  |
| **<**60 | 81 / 1016 | 1.00 (ref) | 41 / 909 | 1.00 (ref) |  |
| 60 to <120 | 96 / 782 | 1.59 (1.16, 2.18) ^f^ | 42 / 635 | 1.37 (0.87, 2.14) |  |
| 120 to <180 | 74 / 506 | 1.91 (1.36, 2.69) ^f^ | 41 / 462 | 1.83 (1.16, 2.89) ^f^ |  |
| **≥**180 | 74 / 406 | 2.41 (1.69, 3.42) ^f^ | 49 / 464 | 1.92 (1.23, 3.01) ^f^ |  |
| p-trend ^d^ |  | <0.0001 ^f^ |  | 0.002 ^f^ | 0.97 |
| **Computer use, m/d** |  |  |  |  |  |
| <60 | 223 / 1908 | 1.00 (ref) | 158 / 2232 | 1.00 (ref) |  |
| 60 to <120 | 48 / 458 | 0.85 (0.61, 1.19) | 11 / 149 | 1.04 (0.55, 1.98) |  |
| **≥**120 | 54 / 344 | 1.28 (0.92, 1.78) | 4 / 89 | 0.50 (0.18, 1.40) |  |
| p-trend ^d^ |  | 0.34 |  | 0.28 | 0.25 |
| **Television watching, m/d** |  |  |  |  |  |
| <60 | 183 / 1698 | 1.00 (ref) | 95 / 1519 | 1.00 (ref) |  |
| 60 to <120 | 79 / 632 | 1.19 (0.90, 1.57) | 46 / 641 | 1.14 (0.79, 1.66) |  |
| **≥**120 | 63 / 380 | 1.60 (1.17, 2.19) ^f^ | 32 / 310 | 1.44 (0.94, 2.22) |  |
| p-trend ^d^ |  | 0.003 ^f^ |  | 0.10 | 0.98 |

Abbreviation: OR, Odds Ratios; CI, Confidence intervals.

^a^ Adjusted for grade (4^th^; 7^th^ grade), parental co-residence (none or one parent; both parents), parents’ highest education (high school or lower; college or higher), monthly household income (<2,000,000; 2,000,000 to <4,000,000; 4,000,000 to <6,000,000; 6,000,000 to <8,000,000; ≥8,000,000 KRW), academic performance (poor or fair; good; missing), and perceived parental control score (continuous).

^b^ Adjusted for sex (boy; girl), parental co-residence (none or one parent; both parents), parents’ highest education (high school or lower; college or higher), monthly household income (<2,000,000; 2,000,000 to <4,000,000; 4,000,000 to <6,000,000; 6,000,000 to <8,000,000; ≥8,000,000 KRW), academic performance (poor or fair; good; missing), and perceived parental control score (continuous).

^c^ P-interaction was estimated using the Wald test for the production term between screen time and grade.

^d^ P-trend was estimated using the Wald test for continuous screen time variables.

^f^ Statistical significance at Bonferroni-corrected α level of 0.0125 (4 exposures)

**Supplementary Figure 1. Flow chart of study participants**

**5,197** Korean students from the Korean Children and Youth Panel Survey 2018 (KCYPS 2018)

(**2,607** in 4^th^ grade students and **2,590** in 7^th^ grade students)

**17** students with missing information on weight or height were excluded

(**13** in 4^th^ grade students and **4** in 7^th^ grade students)

**5,180** final study participants

(**2,594** in 4^th^ grade students and **2,586** in 7^th^ grade students)

**Supplementary Figure 2. Odds ratios (ORs) and 95% confidence intervals (CIs) of obesity prevalence associated with substituting 1-hour of screen time for non-screen time activities among the 4^th^ grade students**

The graphs show the ORs and 95% CIs of obesity prevalence associated with increasing 1-hour of each non-screen time activity while decreasing 1-hour of (A) combined screen time, (B) smartphone use, (C) computer use, and (D) television watching among 4^th^ grade students. Models adjusted for sex (boy; girl), parental co-residence (none or one parent; both parents), parents’ highest education (high school or lower; college or higher), monthly household income (<2,000,000; 2000,000 to <4,000,000; 4,000,000 to <6,000,000; 6,000,000 to <8,000,000; ≥8,000,000 KRW), academic performance (poor or fair; good; missing), perceived parental control (score 4-16, continuous), and all non-screen time activities (performing physical activity, sleeping, hanging out with friends, reading, studying, chatting with parents).

| **(A)** | **(B)** |
| --- | --- |
| **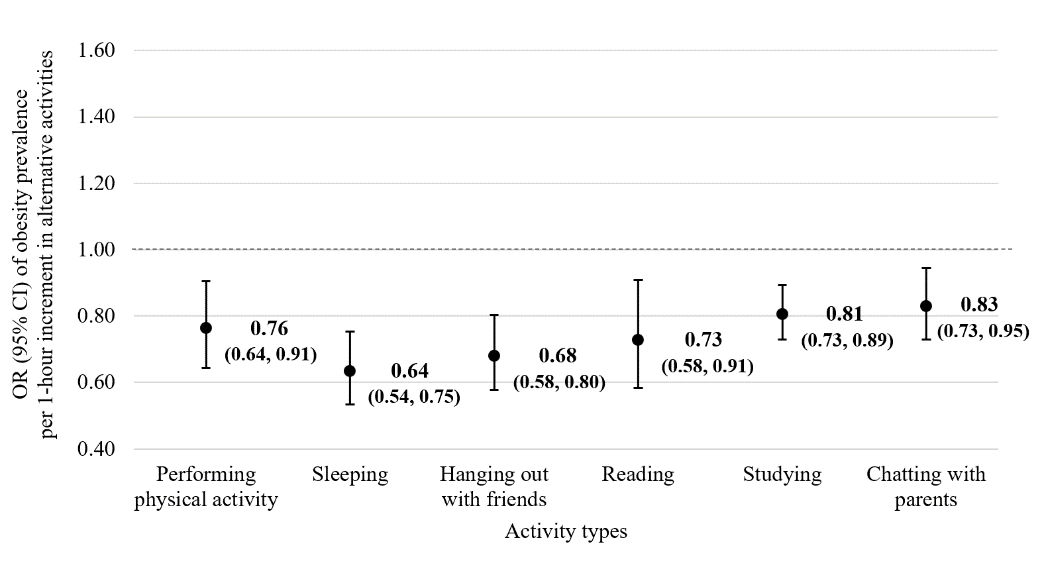** | **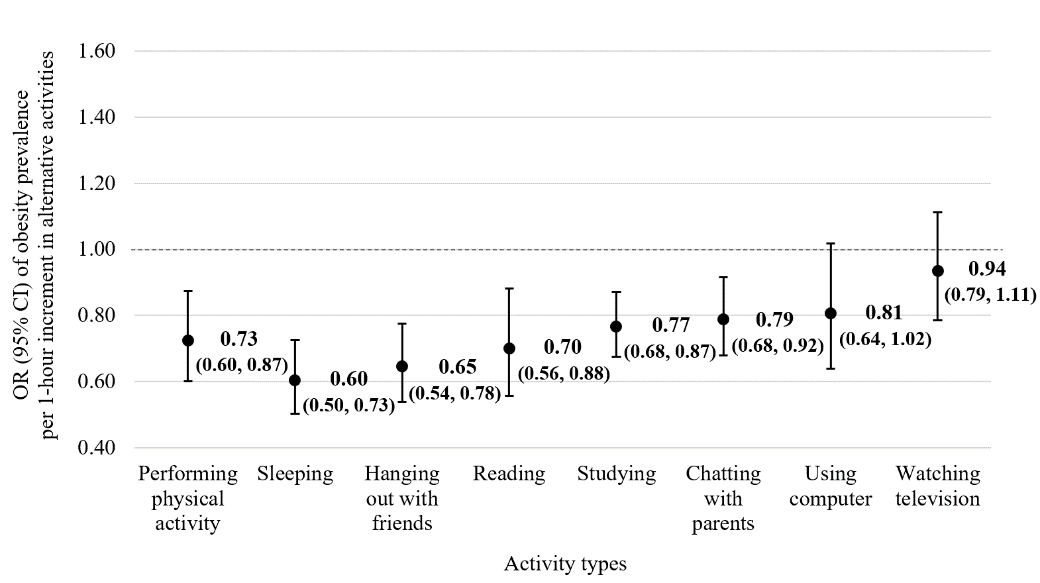** |
| **(C)** | **(D)** |
| **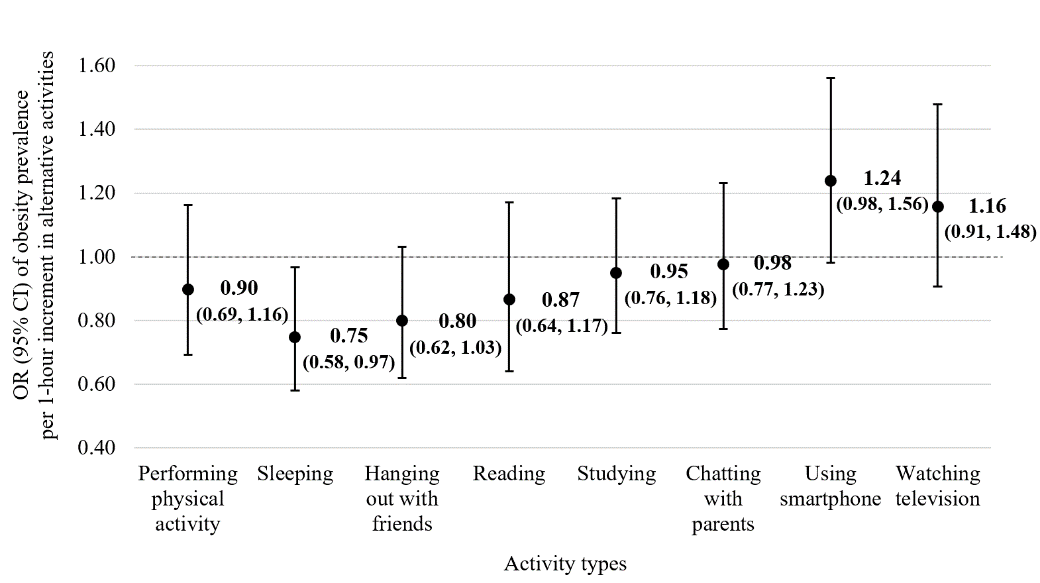** | **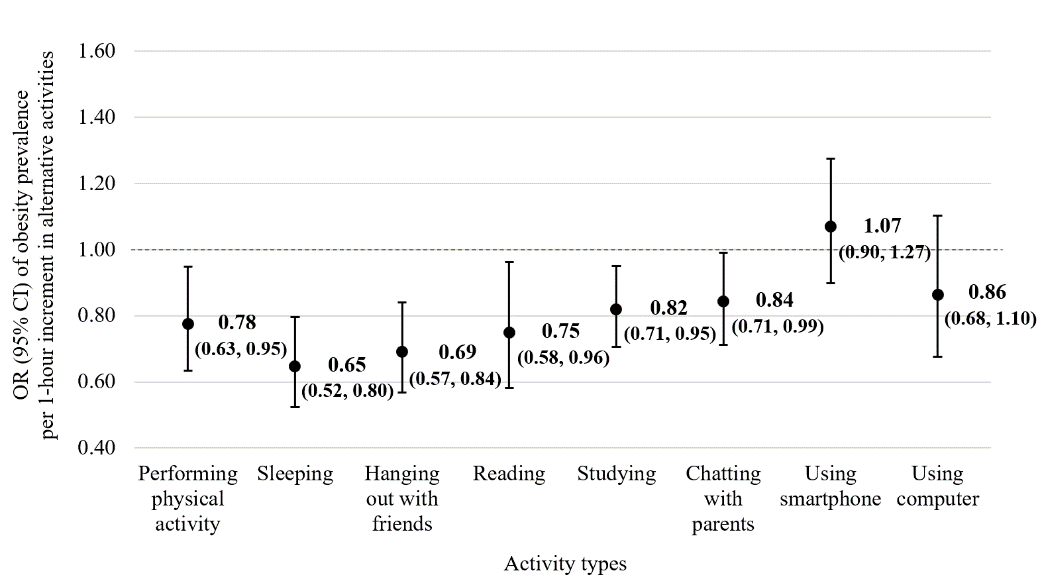** |

**Supplementary Figure 3. Odds ratios (ORs) and 95% confidence intervals (CIs) of obesity prevalence associated with substituting 1-hour of screen time for non-screen time activities among the 7^th^ grade students**

The graphs show the ORs and 95% CIs of obesity prevalence associated with increasing 1-hour of each non-screen time activity while decreasing 1-hour of (A) combined screen time, (B) smartphone use, (C) computer use, and (D) television watching among 7^th^ grade students. Models adjusted for sex (boy; girl), parental co-residence (none or one parent; both parents), parents’ highest education (high school or lower; college or higher), monthly household income (<2,000,000; 2000,000 to <4,000,000; 4,000,000 to <6,000,000; 6,000,000 to <8,000,000; ≥8,000,000 KRW), academic performance (poor or fair; good; missing), perceived parental control (score 4-16, continuous), and all non-screen time activities (performing physical activity, sleeping, hanging out with friends, reading, studying, chatting with parents).

| **(A)** | **(B)** |
| --- | --- |
| **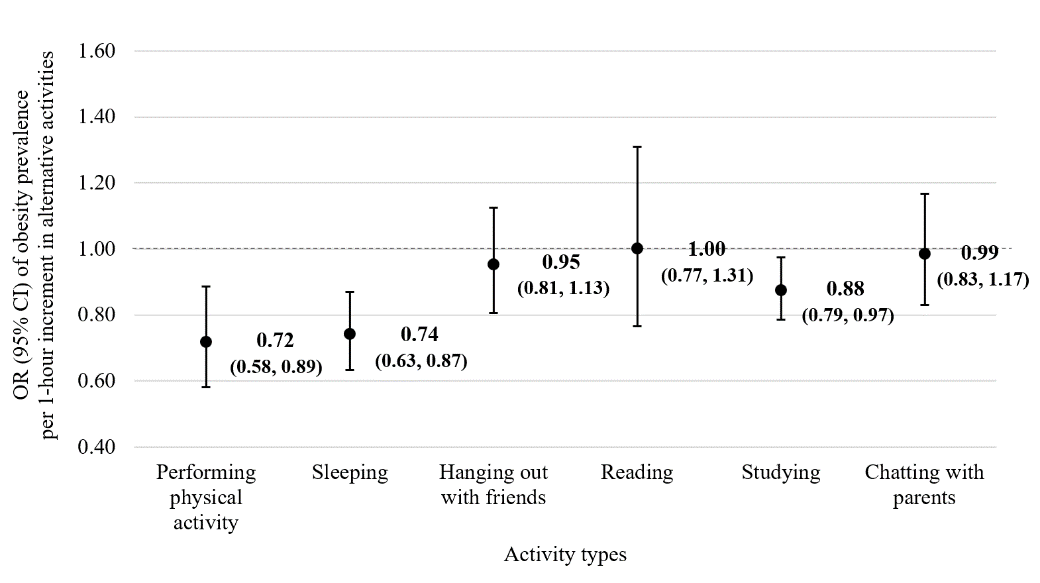** | **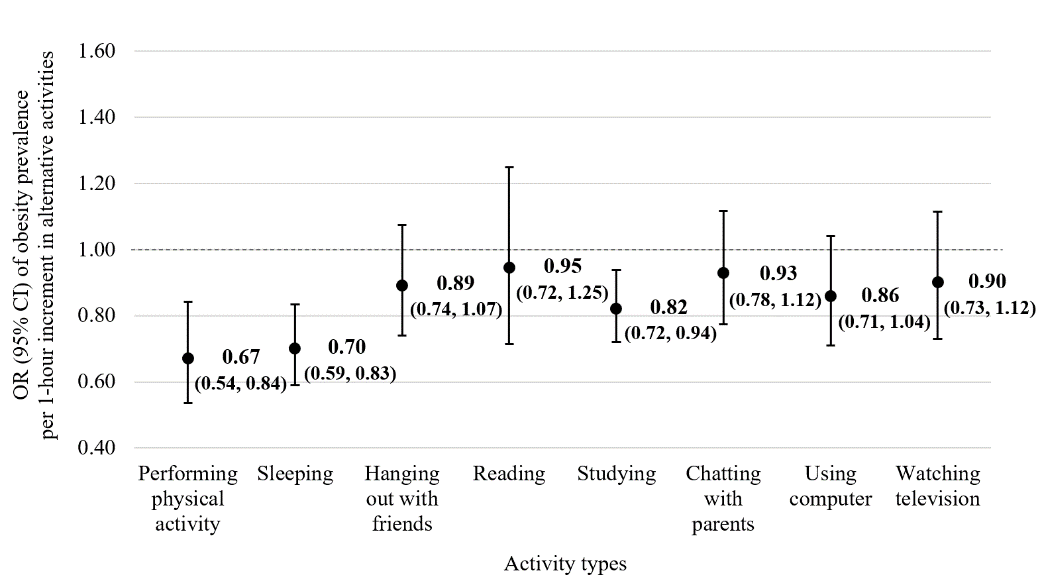** |
| **(C)** | **(D)** |
| **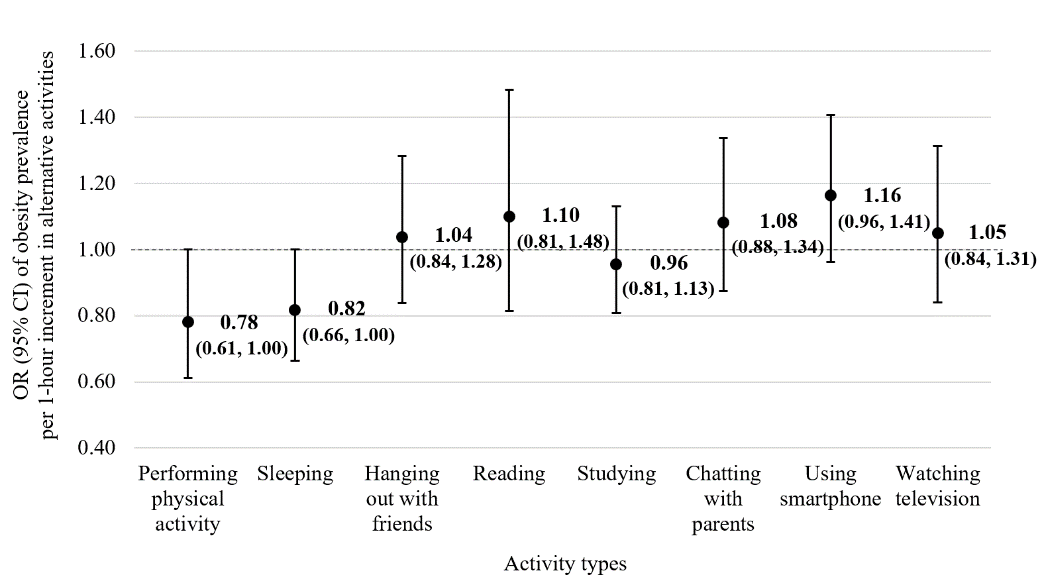** | **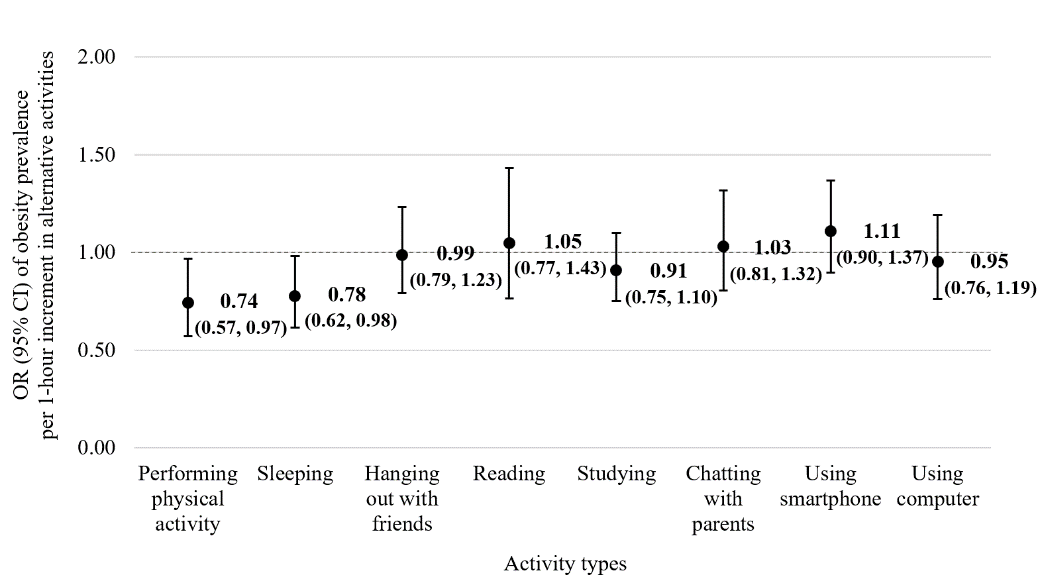** |

**Supplementary Figure 4. Odds ratios (ORs) and 95% confidence intervals (CIs) of obesity prevalence associated with substituting 1-hour of screen time for non-screen time activities among boys**

The graphs show the ORs and 95% CIs of obesity prevalence associated with increasing 1-hour of each alternative activity while decreasing 1-hour of (A) combined screen time, (B) smartphone use, (C) computer use, and (D) television watching among boys. Models adjusted for school grade (4th, 7th grade), parental co-residence (none or one parent; both parents), parents’ highest education (high school or lower; college or higher), monthly household income (<2,000,000; 2000,000 to <4,000,000; 4,000,000 to <6,000,000; 6,000,000 to <8,000,000; ≥8,000,000 KRW), academic performance (poor or fair; good; missing), parental psychological control score (continuous), and all alternative after-school activities (sleeping, performing physical activity, hanging out with friends, chatting with parents, studying, reading).

| **(A)** | **(B)** |
| --- | --- |
| **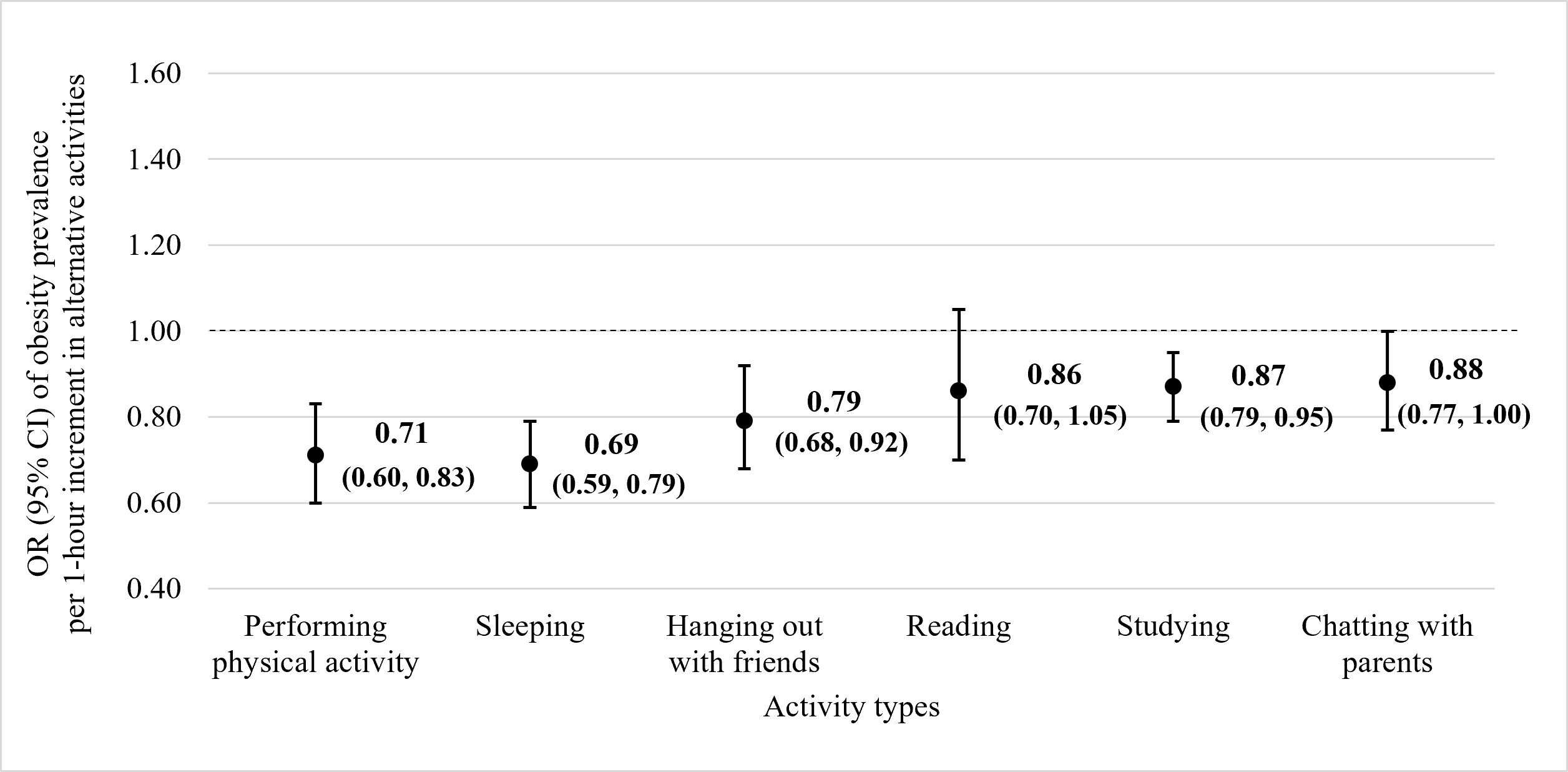** | **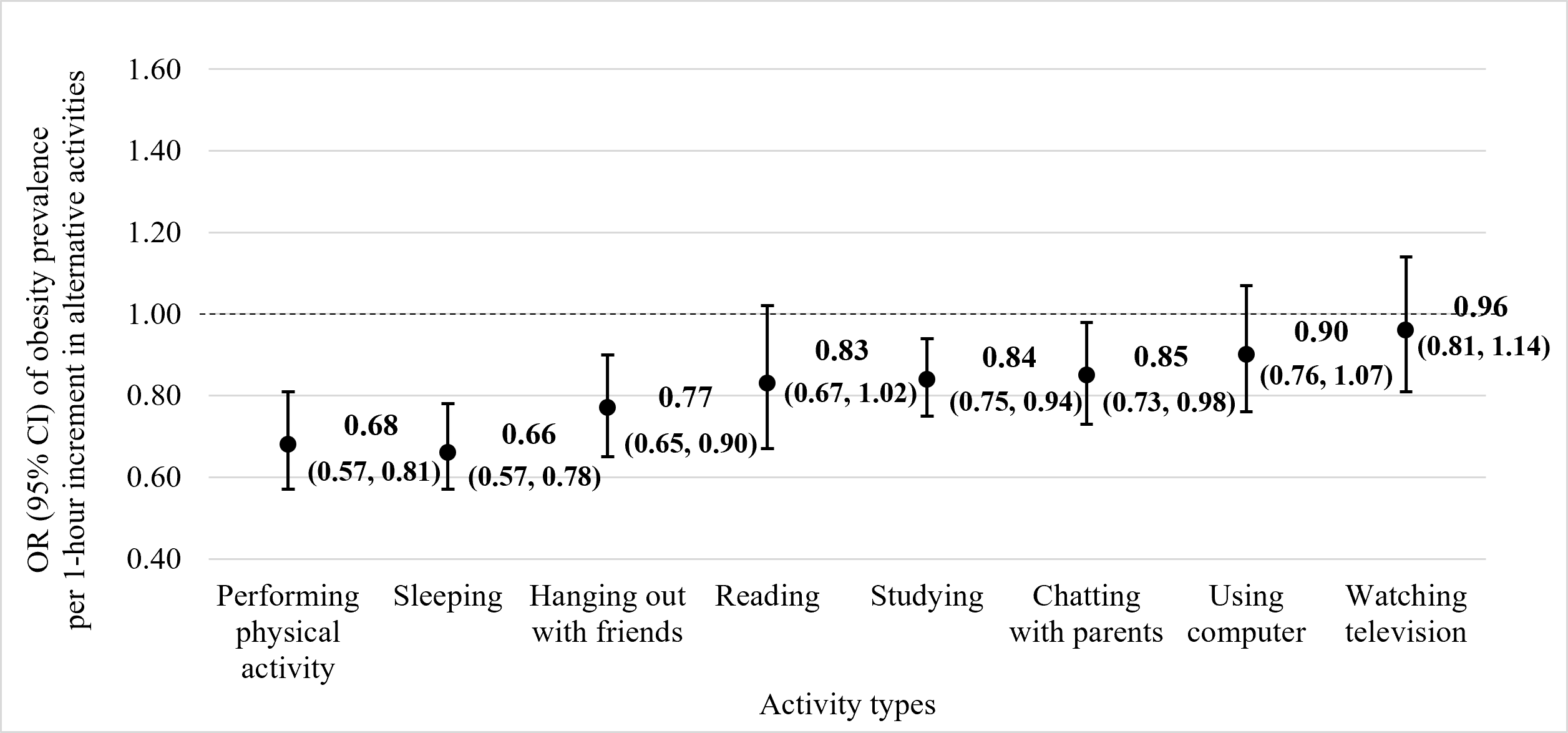** |
| **(C)** | **(D)** |
| **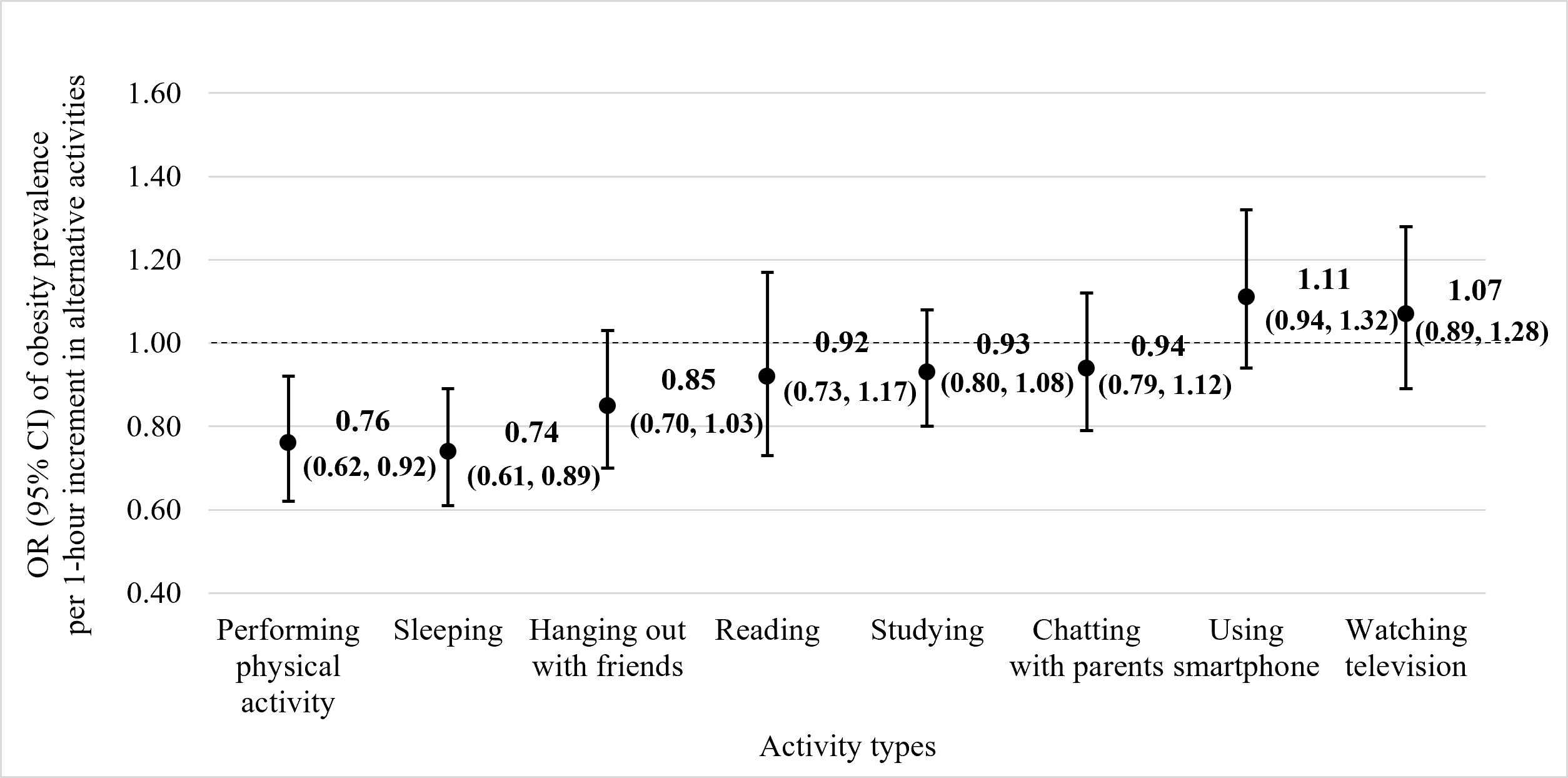** | **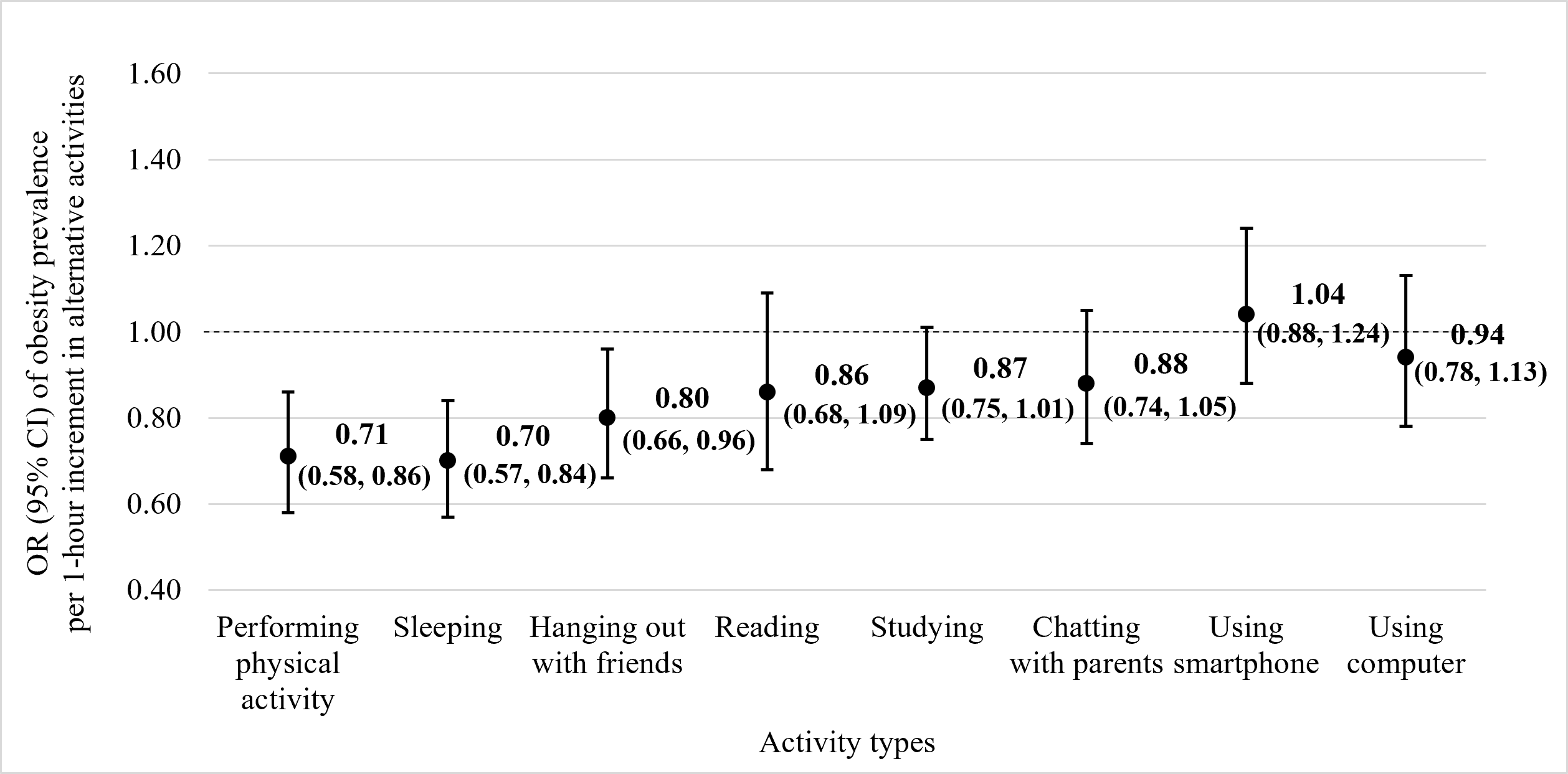** |

**Supplementary Figure 5. Odds ratios (ORs) and 95% confidence intervals (CIs) of obesity prevalence associated with substituting 1-hour of screen time for non-screen time activities among girls**

The graphs show the ORs and 95% CIs of obesity prevalence associated with increasing 1-hour of each alternative activity while decreasing 1-hour of (A) combined screen time, (B) smartphone use, (C) computer use, and (D) television watching among girls. Models adjusted for school grade (4th, 7th grade), parental co-residence (none or one parent; both parents), parents’ highest education (high school or lower; college or higher), monthly household income (<2,000,000; 2000,000 to <4,000,000; 4,000,000 to <6,000,000; 6,000,000 to <8,000,000; ≥8,000,000 KRW), academic performance (poor or fair; good; missing), parental psychological control score (continuous), and all alternative after-school activities (sleeping, performing physical activity, hanging out with friends, chatting with parents, studying, reading).

| **(A)** | **(B)** |
| --- | --- |
| **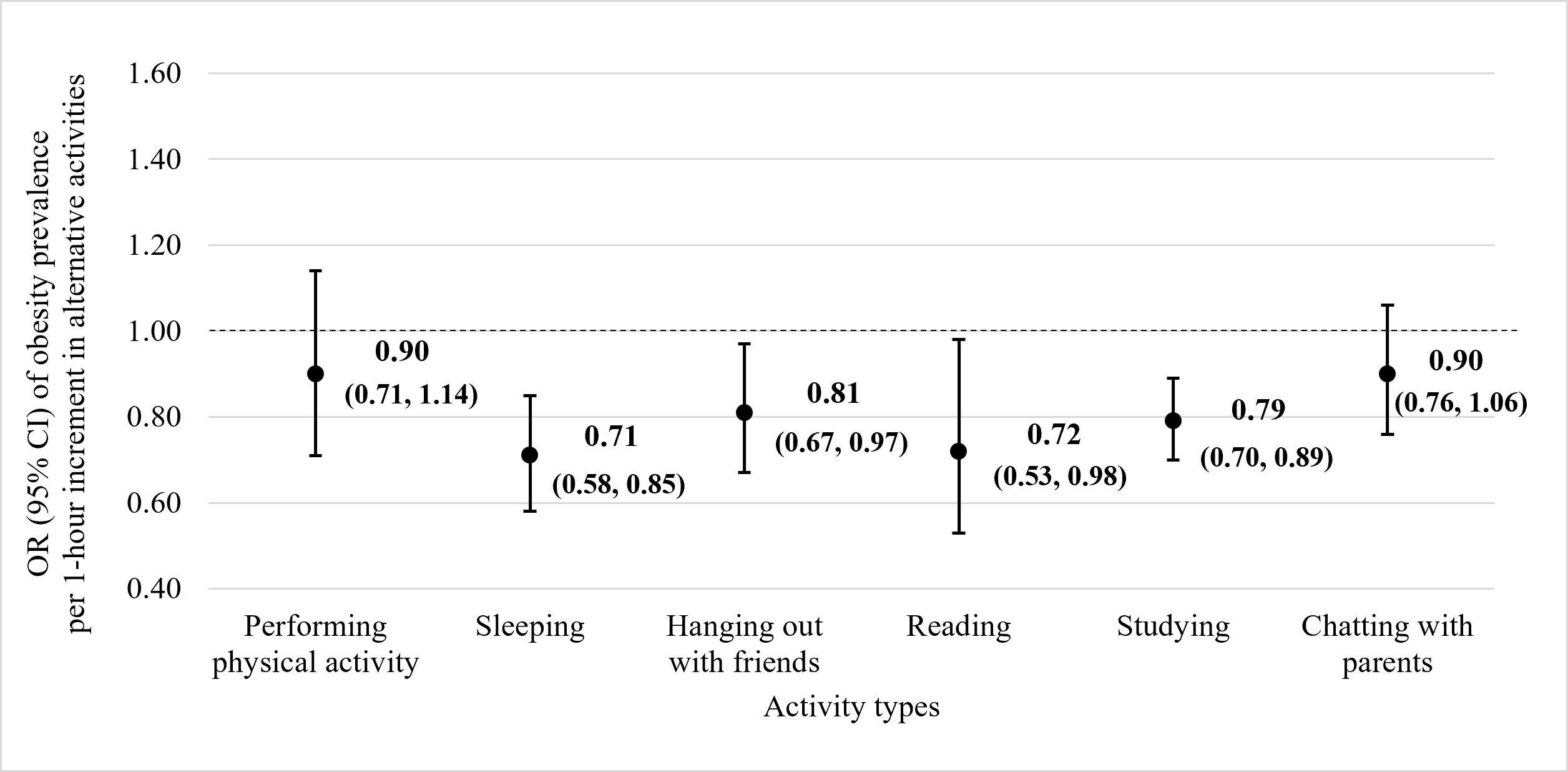** | **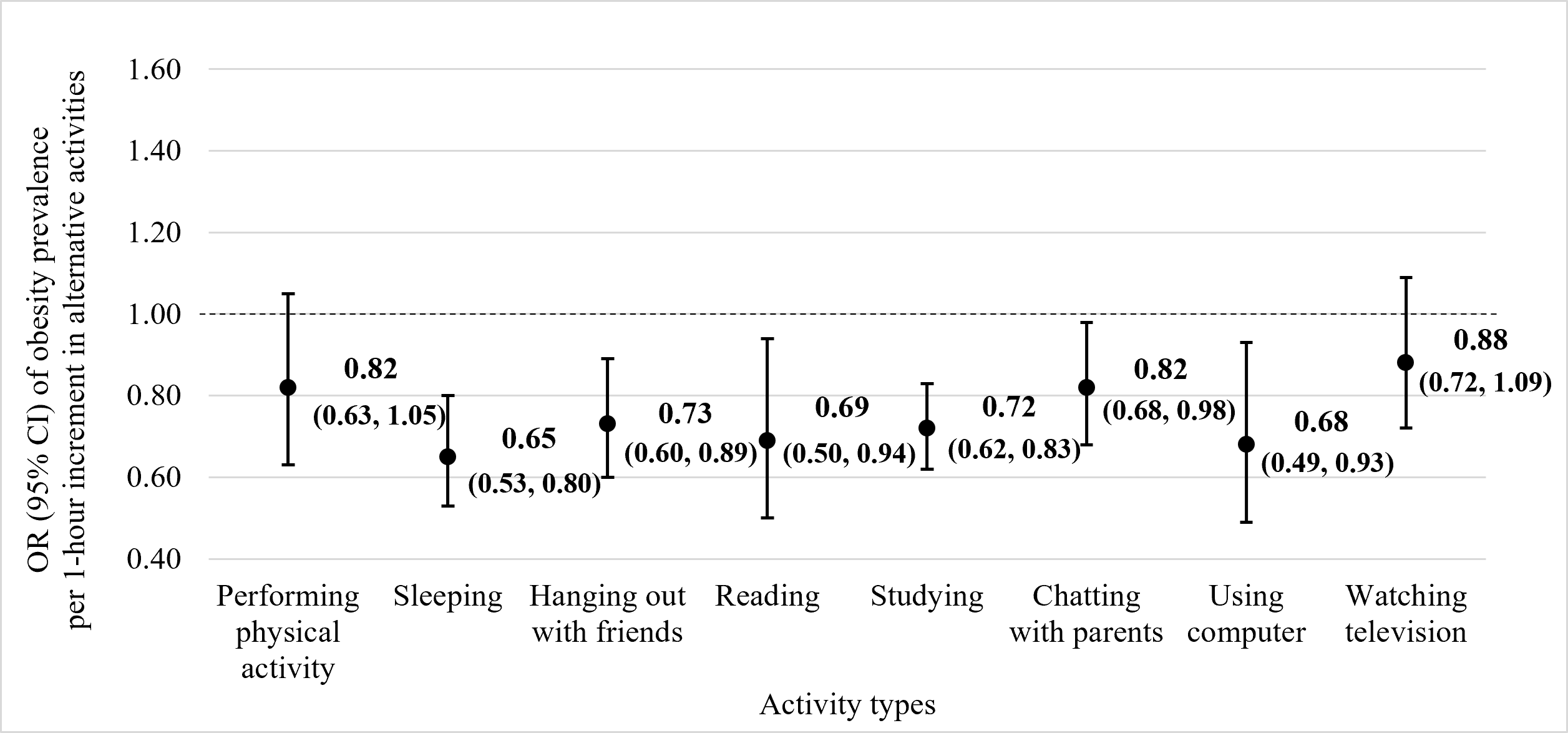** |
| **(C)** | **(D)** |
| **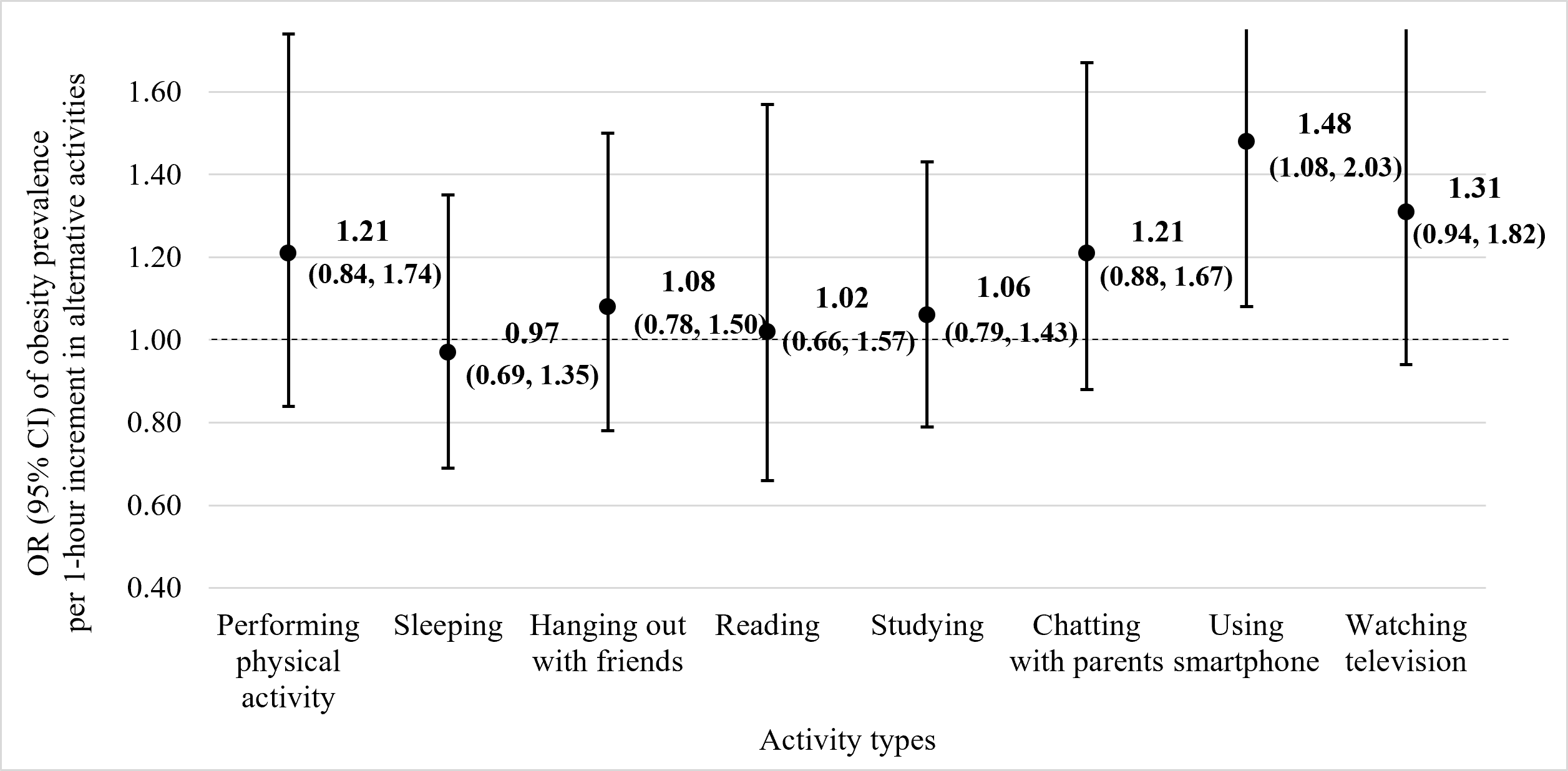** | **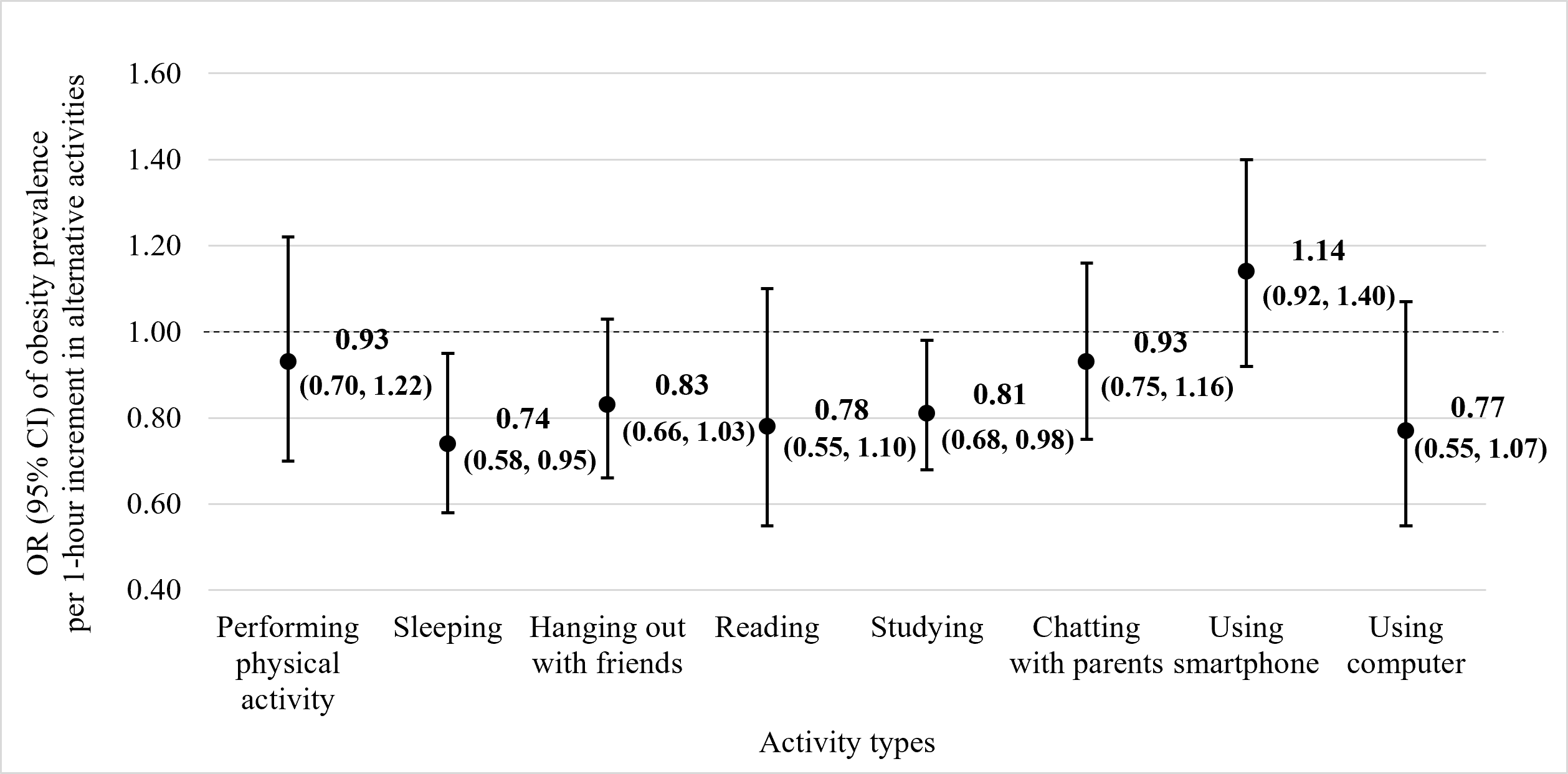** |
